# Initial peptidomic profiling of Brazilian sea urchins: *Arbacia lixula*, *Lytechinus variegatus* and *Echinometra lucunter*

**DOI:** 10.1186/s40409-016-0071-x

**Published:** 2016-05-04

**Authors:** Juliana Mozer Sciani, Andrews Krupinski Emerenciano, José Roberto Machado Cunha da Silva, Daniel Carvalho Pimenta

**Affiliations:** Laboratory of Biochemistry and Biophysics, Butantan Institute, Av. Vital Brasil, 1500, São Paulo, SP CEP 05503-900 Brazil; Laboratory of Evolutionary Histophysiology, Department of Cell and Developmental Biology, Institute of Biomedical Sciences, University of São Paulo (USP), São Paulo, SP Brazil

**Keywords:** Peptides, Toxins, Sea urchin, Peptidomics

## Abstract

**Background:**

Sea urchins can be found throughout the Brazilian coast and are reported to be one of the major causes of marine accidents on the shoreline. Although not lethal, these accidents are reported to be extremely painful. In order to understand the toxinology of the Brazilian urchins, a peptidomic approach was performed aiming to characterize the naturally occurring peptides in both the coelomic fluid and the spine.

**Methods:**

Animals were collected without gender distinction and samples of the coelomic fluid and spines extracted were analyzed by RP-HPLC and mass spectrometry for peptide *de novo* sequencing.

**Results:**

Several peptides were identified either in the coelomic fluid or the spine extract (except for *E. lucunter*). The peptide sequences were aligned with public deposited sequences and possible functions were inferred. Moreover, some peptides can be cryptides, since their sequences were identified within functional proteins, for example thymosin from *Strongylocentrotus purpuratus.*

**Conclusions:**

Although preliminary, the peptidomic approach presented here reports, for the first time, the abundance of novel biological molecules derived from these animals. The discovery of such molecules may be of potential biotechnological application, as described for other organisms; nevertheless, further studies are required.

**Electronic supplementary material:**

The online version of this article (doi:10.1186/s40409-016-0071-x) contains supplementary material, which is available to authorized users.

## Background

Peptides are molecules that fill gaps in current therapeutic approaches, between small organic molecules (<500 Da) and the so-called ‘true’ biologicals (>5000 Da). It is thought that peptides represent a class of potential interesting biotechnological molecules to be developed, regarding their specificity and high potency versus the (relative) small size and lower manufacture costs. Issues regarding their biodistribution and enzymatic degradation had discouraged their development as drugs, but these problems can now be bypassed by using new formulations and/or injectable solutions [1, 2].

Peptides are being assayed for several uses, and clinical trials are being conducted for peptide drugs [1]. The marine biodiversity has already provided molecules with therapeutic potential; therefore, they comprise a good source of molecules that has been poorly explored. Marine animals synthesize/secrete/process molecules for several purposes, including chemical defense against predators, microorganisms and even digestion, constituting a broad arsenal waiting to be explored. Among the already described molecules isolated from marine organisms, terpenoids, alkaloids, steroids and peptides can be included [3].

The mass spectrometry-based investigation of peptides, or peptidome, is a fast and efficient technique to identify peptides present in a given sample. This approach was employed in this work to identify peptides in three Brazilian sea urchin species. These animals are commonly found in Brazil, along the whole coastline. *Echinometra lucunter*, *Lytechinus variegatus* and *Arbacia lixula* are the most abundant species of sea urchins in Brazil.

*E. lucunter* is a widespread species, commonly found in shallow waters (in tide pools and reef slopes) [4]. *A. lixula* and *L. variegatus* are also found in reef slopes, but mainly in the sandy bottom [5]. The composition of their venoms, regarding the molecules actually present in those sea urchin species, has never been not fully described; however, some biological activities have already been reported for *E. lucunter* sea urchin, clearly signaling for the presence of bioactive molecules [6–8].

The aim of the present work was to identify peptides in coelomic fluid and spine extract of three Brazilian sea urchins, and correlate them to already described peptides and respective biological activities.

## Methods

### Sea urchin collection

Sea urchins (*Echinometra lucunter*, *Lytechinus variegatus* and *Arbacia lixula*) were collected in São Sebastião, SP, Brazil, under license number 13852–1 from the Brazilian Institute of Environment and Renewable Natural Resources (IBAMA) and in partnership with the Center for Marine Biology (CEBIMar/USP).

Animals were collected without distinction of gender, age or size. The coelomic fluid (circa 30 mL) was extracted from the sea urchin by puncturing the peristomial membrane. Spines were removed and immersed in a buffered solution (ammonium acetate 100 mM, pH 7.3) for molecule extraction for 24 h, at 4 °C.

### Solid phase extraction fractioning

The coelomic fluid and spine extracts were submitted to solid phase extraction (SPE), in C18 cartridges (Phenomenex) and contents were eluted with 0, 25, 50 and 100 % acetonitrile in ultrapure water, containing 0.1 % trifluoroacetic acid. Fractions were lyophilized and submitted to mass spectrometry analysis.

### Mass spectrometry analysis

Analyses were performed by RP-HPLC coupled to an ESI-IT-ToF mass spectrometer (Shimadzu Co., Japan). Fractions from SPE were loaded into a C18 column (2.1 × 50 mm, 100 A, Phenomenex) and the elution was carried out in gradient mode, with two solvents: (A) ultrapure water containing 0.1 % acetic acid, (B) acetonitrile 90 % in ultrapure water containing 0.1 % acetic acid. The gradient was performed in 0 to 100 % B for 30 min.

After UV detection by HPLC, eluted molecules were automatically analyzed by mass spectrometry, under positive ionization mode. The interface voltage was kept at 4.5 kV, the detector voltage at 1.70 kV and the capillary temperature at 200 °C. The mass range used was 100–2000 m/z. Data dependent acquisition (DDA) MS/MS analysis was performed.

MS and MS/MS data were processed by PEAKS® 7.0 software (Bioinformatics Solution Inc.) and manually checked for accuracy and correctness. *De novo* sequences were searched against peptide databank (PepBank) [9]. In addition, a database of peptides was manually constructed with published data from other sea urchins species [10–13]. These described peptides were aligned with the *de novo* sequences by Clustal Omega multiple sequence alignment, with output format without numbers, mBed-like clustering guide-tree and iteration.

## Results

**Fig. 1:**
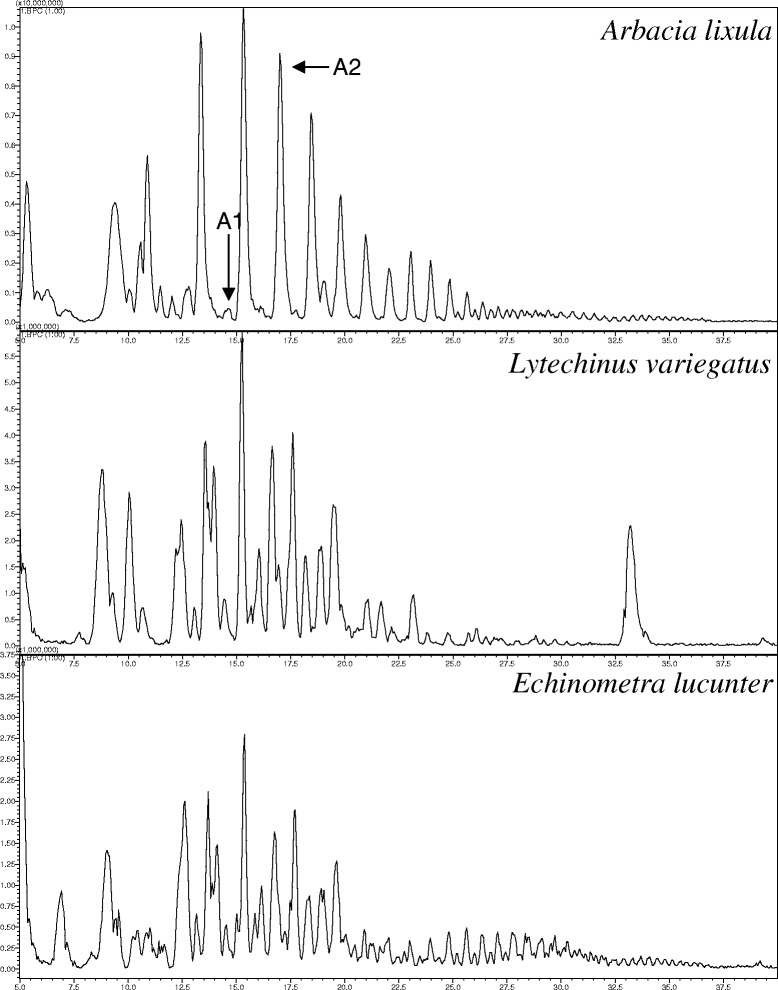
TIC chromatograms of the RP-HPLC analyses of coelomic fluid from 25 % SPE of three sea urchins. A1 and A2 were selected as representative peaks to which complementary analyses are presented in Fig. 3

**Fig. 2:**
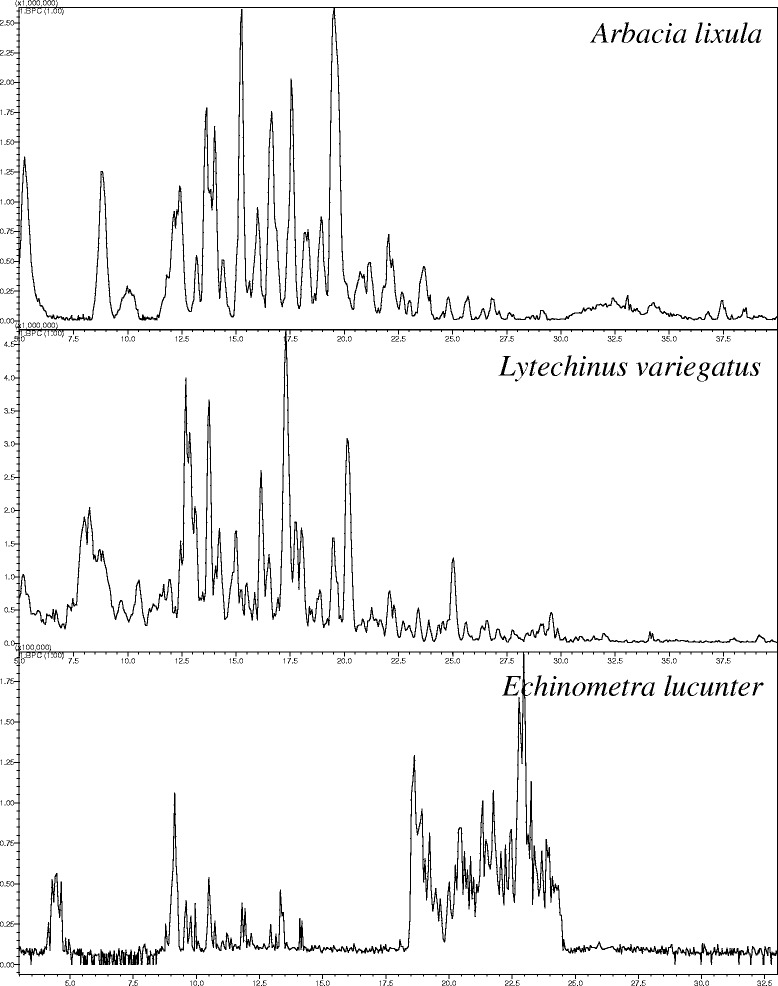
TIC chromatograms of the 50 % SPE fractions of sea urchin spine extracts

**Fig. 3:**
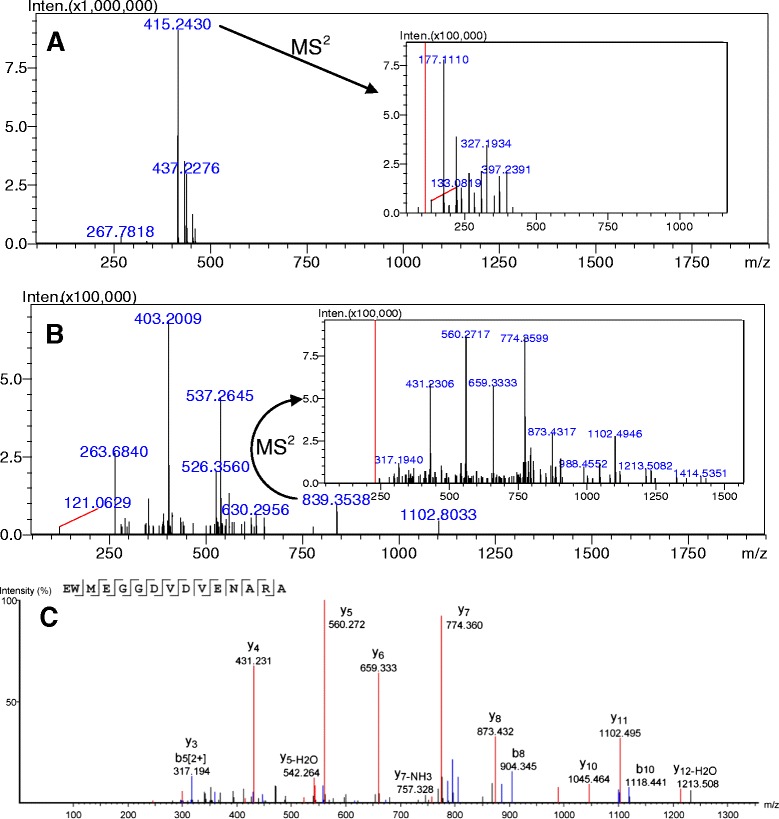
**a** MS^1^ and MS^2^ (inset) spectra of A2 [Fig. 1]. **b** MS^1^ and MS^2^ (inset) spectra of A1 [Fig. 1]. **c** PEAKS® Studio processed m/z 839.35 MS^2^ spectrum with the deduced sequence above the annotated peaks

Figures 1 and 2 are the representative total ion chromatogram (TIC) for SPE extractions of the biological samples analyzed in this study. Figure 1 contains the TIC chromatograms of the RP-HPLC analyses of the 25 % SPE of coelomic fluid from three sea urchins. A1 and A2 were selected as representative peaks to which complementary analyses are presented in Fig. 3. Figure 2 contains the TIC of the 50 % SPE fractions of sea urchin spine extracts. It is noteworthy to mention the differences between the species and the low abundance and poor composition of *E. lucunter* spine extract, as previously reported [8].

Figure 3 contains two representative analyses of distinct peaks selected from Fig. 1 (A1 and A2) based solely on their relative abundance, but bearing similar hydrophobicity, e.g. RP-HPLC retention times. Figure 3, panel A, contains the MS^1^ and MS^2^ spectra of A2, the second largest TIC peak in *A. lixula* chromatogram. It is clear from the low m/z (415.24; z = 1) and fragmentation pattern that this molecule is not a peptide. This was the case for the vast majority of the larger peaks for all processed biological samples from all animals. Figure 3, panel B, contains the MS^1^ and MS^2^ spectra of A1, one of the several minor TIC peaks detected in *A. lixula* chromatogram. One can observe that, besides being a more complex RP-HPLC fraction, some higher m/z values could be detected. The 839.35 (z = 2) ion was selected and its MS^2^ is presented in an inset. Simple visual inspection of the daughter ions indicates the peptidic nature of the precursor molecule. This spectrum was processed by PEAKS® Studio 7.0 and manually checked for accuracy and precision, as presented in Fig. 3, panel C. This whole rationale was employed for all peaks, from all chromatograms of the three different animals, yielding the many peptides presented in Tables 1 and 2.

**Table 1:** Peptides identified and *de novo* sequenced in coelomic fluid of sea urchins

*Arbacia lixula*	*Lytechinus variegatus*	*Echinometra lucunter*
DVKL	FLSY	LGSR
FLCF	LFLSE	LLHA
LDLR	GHNGY	LPPP
LLLL	STLEP	PPVF
MECR	AALTPE	SCSM
NLVM	ASAGAH	VFMA
PESL	MLGGAH	LASVP
LSDCL	VDSAHA	LGQLT
LAPAA	AARFGLR	VTTKH
LVTELL	TVTLAGAH	TGGGLPV
DGHCGAD	DTFAQLPEAEP	REGSPDLR
HSGECSF	LVVDNGSGFLN	VEGSLVLR
VTLMMSS	VNDGTAALVVDNGSGFKV	FLMLVDGHN
FVKVEVLPQ		FPVAHPYGVQA
REGSVCVEH		AAPCPDVEVSEQF
VAKGSPDLNK		EHEAATLVVLQPE
FVEQVLVEPQ		PSVGVVTLPTELPQ
MTVNGASVTN		PLTPTSVESVDPLPQ
GDKGSTAGSNH		QPMVVLCLVSTFDKSK
MTKYAATGVTN		EVKPDDVESASHGPLS
TSTLLFDAHVT		EDGAPDVSEVGGTFDQ
EDQLVVKEVETF		SGPMPDVSEGFGASLMQ
APAEYLPVELPLYWY		
EWMEGGDVDVENARA		
KTGGGGVSGGSAGDH		
YMAAGASSSSSTKVVQK

**Table 2:** Peptides identified and *de novo* sequenced in the spines of sea urchins

*Arbacia lixula*	*Lytechinus variegatus*	*Echinometra lucunter*
GLAH	AAHE	<−>
NLVM	GAGN	
NRDT	LLHA	
TAAH	LVAL	
FLMEV	SHPW	
LLGHH	VTTKH	
TSLEP	TAEGAH	
DTGSAD	VDSAHA	
ECNGPGW	LATVTEH	
ELMVVC	VNDLLYTN	
MAAPSD	FEDLMLPGLL	
MTSKPTW	QPLGAATLVVLEQP	
REGSPDLR		
GLKGSPDLR		
REGSPDLLE		
TNLLGTGAH		
MQLFVGSNLE		
PYLFGGMLQL		
FPGCFYPGANL		
LDAEDLETFKL		
EEEVAALVVDNGSGMVK		
NCVATSGGDVDVEAGGRA		
LLAGSGAGGGPNVEDADPEPK		

Even though the direct proteomic approach (e.g., spectrum matching) yielded no results in the public databases (NCBI), several *de novo* sequences of peptides could be obtained for the three analyzed species, in both spines (except for *E. lucunter*) and coelomic fluid as shown in Tables 1 and 2, respectively, and in Additional file 1. *Arbacia lixula* seemed to contain more peptides than the other two species, in spines and coelomic fluid, whereas *Lytechinus variegatus* possess an equivalent number of peptides in coelomic fluid and spines. For *Echinometra lucunter* it was not possible to retrieve peptides from the spines – only in coelomic fluid.

These sequenced peptides were then checked against a peptide database (PepBank) and some similarities could be identified, as listed in Table 3. Interesting biological activities were found for the matched sequences, such as antibiotic, antitumor, phospholipase A_2_ inhibitors or neuroprotective. A similarity was also found with a toxin from scorpion venom, which confirms the presence of toxins in sea urchins, as recently described by our group [6, 7].

**Table 3:** Search of *de novo* sequenced peptides of sea urchins in PepBank

Peptide	Origin	Function (possible)
DVKL	*A. lixula* – coelomic fluid	Antibiotic and anticancer
FLSY	*L. variegatus* – coelomic fluid	Phospholipase A_2_ inhibitor
LDLR	*A. lixula* – coelomic fluid	Peptide in thrombin-liberated human/eglin (inhibitory activity towards human leukocyte elastase, cathepsin G, porcine pancreatic elastase and alpha-chymotrypsin)
LGSR	*E. lucunter* – coelomic fluid	Brain peptide for activation of prothoracic gland, factor VIII activity, toxin Tc61 (scorpion), human neutrophil NADPH oxidase factor 2
LLLL	*A. lixula* – coelomic fluid	Antimicrobial, neuroprotective
LPPP	*E. lucunter* – coelomic fluid	Dipeptidyl carboxypeptidase substrate
LVAL	*L. variegatus* – spines	Antiviral
PESL	*A. lixula* – coelomic fluid	Membrane-bound aminopeptidase P, protein tyrosine kinases

Moreover, *de novo* sequenced peptides were aligned with other described peptides from sea urchins, especially *Strongylocentrotus purpuratus*, the most studied species. Some peptides have similar sequences, as shown in Additional file 2, with thymosin, halocyntin papillosin, centrocin, AjANP1, AjANP2, AjPPLN2a and other neuropeptides.

## Discussion

Peptides from natural sources are gaining increasing attention as drug leads. In this regard, marine animals have an arsenal of molecules, only a few explored in the drug development field.

Peptides from marine animals have already been described, such as those from cone snails and sea anemones. *Conus* venom is rich in peptides, called conotoxins, found in several species. Conotoxins generally bind to ionic channels as agonists or antagonists, causing a paralyzing effect on prey [14, 15]. Despite the toxic effects of the venom, one peptide was isolated, tested for analgesic activity and showed good pharmacological effects and low toxicity. It is currently under clinical trials for chronic pain treatment [16].

Considering the potential of peptides as drug leads and the fact that marine animals are a rich source of such molecules, peptidomic studies were performed in Brazilian sea urchin species in order to prospect new peptides. To the best of our knowledge, this is the first time these animals are studied from this perspective.

Although few peptides had been previously reported from sea urchin samples, our group recently described a peptide (echinometrin) from the coelomic fluid of *Echinometra lucunter* sea urchin, which was selected based on biological-driven assays. The eight-residue peptide (LRKLMLQR) is active over mast cells, promoting histamine release and, consequently, arising an inflammatory reaction in mice [6]. However, in the present work – in which high-throughput DDA *de novo* sequencing peptidomic approach was performed – echinometrin was not identified, not only because it is present in low concentrations, but also because of its low hydrophobicity index, e.g., low C18-RP-HCPL retention time. Moreover, the analytical LC-MS/MS method employed in the current study differed from the MS analysis regarding the first 5 min of chromatographic separation, since no buffer or residual proteomic reagents (such as IAA, DTT, urea) were injected into the mass spectrometer, which causes the loss of extremely hydrophilic molecules. Nevertheless, several other peptides (*n* = 22) present in *E. lucunter* coelomic fluid could be identified. Some of them are related to marine known peptides, for instance PPVF, which is part of SpurS1, a neuropeptide from *Strongylocentrotus purpuratus* [10]. LGSR and LPPP are similar to neuropeptides that act on prothoracic glands or could be internal peptides of factor VIII, scorpion toxin Tc61, human neutrophil NADPH oxidase factor 2 or dipeptidyl carboxypeptidase.

Some of the peptides described in the present study could be aligned with others from sea urchins, which suggests the presence of bioactive peptides in Brazilian sea urchins. For example, antimicrobial peptides were aligned, indicating the presence of peptides with this function, which are present in many animals, especially marines, due to the environment rich in microorganisms [17, 18].

EDGAPDVSEVGGTFDQ, PSVGVVTLPTELPQ and QPMVVLCLVSTFDKSK were aligned with thymosin from *S. purpuratus* (Additional file 2). Thymosin acts on actin filament organization by sequestering actin monomers. Based on our previous studies on peptide toxins, it is our understanding that thymosin may be a source of cryptides that are bioactive peptides generated proteolytically from a non-precursor protein, by non-classical processing enzymes, and that display biological activities related or unrelated to the original protein [19]. The fragments of thymosin described herein were sequenced from secretions, not from cell lysates (e.g., intracellular). Moreover, these three peptides are derived from one single molecule, which indicates a possible ‘natural’ cleavage (not processing or artifact) generating peptides whose effects could be different from the original one (actin organization). For example, Schillaci et al. [11] have already reported that thymosin fragments, from *S. purpuratus,* possess antimicrobial activity. Nevertheless, complimentary experiments are necessary to evaluate this hypothesis.

Spines have also been reported as a potential source of bioactive molecules, since biological effects had been observed in spine extracts. Briefly, an inflammatory reaction in mice was described for the spine extract of *Echinometra lucunter*, confirming the presence of toxins in these structures [8]. Moreover, our group could identify a cathepsin B/X activity in spines, indicating the presence of enzymes for either chemical defense of the animal or tissue remodeling after injury, a frequent phenomenon observed in our collections [7].

In the same study from 2011, we have observed that spines are rich in small molecules, but no peptides are present [8]. These results were corroborated by the peptidomic approach, presented in the current work, in which we could not identify peptides in spines of *Echinometra lucunter.*

*Lytechinus variegatus*, on the other hand, presented peptides in both the spines and coelomic fluid. Although a few molecules have been described, biological effects have been related to this species, indicating the presence of active molecules. In 1963, Mendes et al. [20] reported an acetylcholine-like activity in the pedicellaria of *L. variegatus*, and that this molecule was deactivated when heated or submitted to NaOH, a situation typical for peptides or proteins. Nevertheless, this molecule (or molecules) has never been biochemically characterized.

In fact, several biological activities can be related to *L. variegatus* peptides obtained by *de novo* sequencing. FLSY, for example, is a phospholipase A_2_ inhibitor and LVAL has antiviral activity. Moreover, AAHE was aligned with centrocin1a, an antimicrobial peptide from *Strongylocentrotus droebachiensis*; whereas VNDGTAALVVDNGSGFKV was aligned with papilosin, and GHNGY and MLGGAH were aligned with halocyntin [12, 13]. Papilosin and halocyntin are antimicrobial peptides obtained from hemocytes of the red sea squirt *Halocynthia papillosa*. These cells, responsible for phagocytosis and involved in the immune system, are also find in abundance in coelomic fluid of sea urchins [13]. Peptides similar to papilosin and halocyntin were identified in coelomic fluid of *L. variegatus*, which indicates the presence of antimicrobial molecules that are essential for maintenance of “sterile” conditions.

*Arbacia lixula* was the sea urchin with the largest number of identified peptides, both in spines and in coelomic fluid. From the latter, the sequenced peptides related to already described ones (not only marine) matched: an antimicrobial peptide, a peptide liberated by thrombin, inhibitory peptides towards human leukocyte elastase, cathepsin G, porcine pancreatic elastase, alpha-chymotrypsin, neuroprotective peptides, membrane-bound aminopeptidase P and protein tyrosine kinases fragments.

Since *L. variegatus* centrocin 1a and 2 also matched sequenced peptides from coelomic fluid of *A. lixula,* it is our opinion that antimicrobial peptides would be present in this sea urchin species. Corroborating this idea, halocyntin was also found in *A. lixula*, and interestingly, in both coelomic fluid and spines. Moreover, there was also matching with papillosin, another antimicrobial peptide.

## Conclusions

This work reported, for the first time, the presence of peptides on the coelomic fluid and spines of three different species of Brazilian sea urchins. By sequence comparison and alignment, some biological activities, especially antimicrobial, could be inferred.

Currently, several peptides are being used as drug leads and other biotechnological applications, so the discovery of new structures could be a starting point for future drug development. Here, we present the Brazilian sea urchins as potential sources of novel peptides, and this initial biochemical characterization may lead to a better understanding of the chemical ecology of the animal, as well as unveil potential biotechnological application for this marine organism.

## Additional files

Additional file 1: List of *de novo* sequenced peptides by PEAKS® software. (CSV 12 kb) Additional file 2: Alignment of peptides obtained from *E. lucunter*, *L. variegatus* and *A. lixula* with the peptides described from other sea urchin species. (DOCX 18 kb)
