# Supplementary material for: Initial peptidomic profiling of Brazilian sea urchins: Arbacia lixula, Lytechinus variegatus and Echinometra lucunter
Source: J Venom Anim Toxins Incl Trop Dis. 2016 May 4;22:17. doi: 10.1186/s40409-016-0071-x (PMC4857251; doi:10.1186/s40409-016-0071-x)
Supplement: Additional file 2: — Alignment of peptides obtained from E. lucunter, L. variegatus and A. lixula with the peptides described from other sea urchin species. (DOCX 18 kb) [file 40409_2016_71_MOESM2_ESM.docx]

**Supplementary material 2**

**REGSPDLR**

halocyntin -FWGHI----WN---AVKRV----GANALHG--AVTGALS-----

peptide ------------------RE----GSPDLR---------------

papillosin GFWKKVGSAAWGGVKAAAKGAAVGGLNALAK--HIQ---------

**PPVF**

peptide -------------------PP-V-----F--------------

neuropeptide -------------------PP-VTYRSKFTF------------

neuropeptide --------------------G-Y---SPFMF------------

neuropeptide ----------------------FK--SPFMF------------

neuropeptide -------------------PP-FK--SPFMF------------

**REGSPDLLE**

halocyntin ------------------------RVGAN----ALHGAVTGALS-

peptide ------------------------REGSP----DLLE--------

**FLCF**

neuropeptide -----------------------SGPYSFNSGLTF------------

peptide ----------------------------F---LCF------------

neuropeptide -----------------------SG---Y-SVLYF------------

**MTKYAATGVTN**

thymosin -MADKPDVSEVASFDKSKLKKAETQEKNTLPTKETIEQEKTA

peptide ------------------------MTKYAATGVTN-------

papillosin --------GFWKKVGSAAWGGVKAAAKGAAVGGLNALAKHIQ

**LDLR**

peptide -----------------------------LD-LR---------

centrocin 2 SWFSRTVHNVGNAVRKGIHAGQGVCSGLGLD-LRAICAGAHAL

centrocin 1a GWFKKTFHKVSHAVKSGIHAGQRGCSALGFD-LRGACAAAHAL

**DTENVREF**

AjANP2 ------------------ANR------YNALR-----------

AjPPLN2a ----------------------FGNSNMDPLVH--SLIG----

peptide -----------------------DTENVREF------------

**FEDLMLPGLL**

thymosin -MADKPDVSEVASFDKSKLKKAETQEKNTLPTKETIEQEKTA

peptide ------------------------FEDLMLPG--LL------

AjPPLN2a -----------------------FGNSNMDPLVHSLIG----

**LVAL**

peptide --------------------------------LVAL---------

papillosin ---GFWK----KVGSAAWGGVKAAAKGAAVGGLNALAKHIQ----

AjANP2 -----------------------ANR------YNALR--------

halocyntin ----FWG----HIWN-------AVKR----VGANALHGAVTGALS

**VAKGSPDLNK**

thymosin -MADKPDVSEVASFDKSKLKKAETQEKNTLPTKETIEQEKTA

peptide VAKGSPDLNK--------------------------------

**GDKGSTAGSNH**

thymosin MADKPDVSEVASFDKSKLKKAETQEKNTLPTKETIEQEKTA----

peptide -----------------------GDKGSTAGSNH-----------

**EDQNAALVVDNGSGVMK**

peptide -----------------------EDQNAAL----VVDNGSGVMK

AjANP1 -------------------------ANRRF----SV--------

**KLPLLQ**

halocyntin -----FWGHI----WN---AVKRVGANALHG---AVTGALS-----

papillosin ----GFWKKVGSAAWGGVKAAAKGAAVGGLN---ALAKHIQ-----

AjANP2 --------------------ANR------YN---ALR---------

peptide ----------------------------KL----PLLQ--------

AjPPLN2a ------------------------FGNSNMD---PLVHSLIG----

thymosin --MADKPDVSEVASFDKSKLKKAETQEKNTL---PTKETIEQEKTA

**LLPK**

centrocin 1a GWFKKTFHKVSHAVKSGIHAGQRGCSALGFDLRGACAAAHAL

centrocin 2 SWFSRTVHNVGNAVRKGIHAGQGVCSGLGLDLRAICAGAHAL

thymosin -MADKPDVSEVASFDKSKLKKAETQEKNTLPTKETIEQEKTA

peptide ----------------------------LLPK----------

papillosin -GFWKKVGSAAWGGVKAAAKGAAVGGLNALAKHIQ-------

**NLVM**

halocyntin ----------FWGHIWNAVKR---VGANALH--GAVTGALS-

peptide ---------------------------N-L-----VM-----

papillosin -GFWKKVGSAAWGGVKAAAKGAAVGGLNALA--KHIQ-----

AjANP2 -----------------ANR------YNALR-----------

**SFTFD**

neuropeptide -----------------------AQPS-F----AF------------

neuropeptide ----------------------GLMPS-F----AF------------

peptide --------------------------S-F----TFD-----------

CiTK2 --------------------SIGDQPSIFNERASFTGLM--------

neuropeptide --------------------------PPFKSPFMF------------

neuropeptide ------------------------PPVTYRSKFTF------------

**PYLFGGMLQL**

peptide --------------------------PYLFGGMLQL------------

AjPPLN2a -------------------FGNSNMDPLVHS-LIG-------------

neuropeptide -------------------SG---YSVLYF------------------

neuropeptide --------------------G---FSKLYF------------------

**LNNDL**

peptide ---------LNN---------------------DL----------

centrocin 2 ---SWFSRTVHNVGNAVRKGIHAGQGVCSGLGLDLRAICAGAHAL

centrocin 1a ---GWFKKTFHKVSHAVKSGIHAGQRGCSALGFDLRGACAAAHAL

**PLTPTSVESVDPLPQ**

peptide ----------------------PLTPTSVES-VDPLPQ----------

AjANP1 -------------------------ANRRFS-V---------------

AjPPLN2a -----------------------FGNSNMDPLVHSLIG----------

**MAAPSD**

neuropeptide ----------------------AQPS-----FAF------------

neuropeptide ---------------------GLMPS-----FAF------------

peptide ---------------------MAAPS-----D--------------

CiTK2 -------------------SIGDQPSIFNERASFTGLM--------

AjANP1 ----------------------ANRR-----FSV------------

**DTFAQLPEAEP**

thymosin -MADKPDVSEVASFDKSKLKKAETQEKNTLPTKETIEQEKTA

peptide ------------------------DTFAQLP---EAEP----

**EVKPDDVESASHGPLS**

halocyntin -------FWGHI----WN---AVKRVGANALHGAVTGALS-------

papillosin ------GFWKKVGSAAWGGVKAAAKGAAVGGLNALAKHIQ-------

AjANP2 ----------------------ANR------YNALR-----------

peptide --------------------EVKPDDVESASHGPLS-----------

neuropeptide ------------------------GDLAF----AF------------

neuropeptide ------------------------G---FNSALMF------------

neuropeptide ------------------------------SALMF------------

neuropeptide ---------------------------GY-SPFMF------------

neuropeptide --------------------------PPFKSPFMF------------

neuropeptide ------------------------PPVTYRSKFTF------------

neuropeptide ------------------------SG---YSVLYF------------

centrocin 1a -----GWFKKTFHKVSHAVKSGIHAGQRGCSALGFDLRGACAAAHAL

centrocin 2 -----SWFSRTVHNVGNAVRKGIHAGQGVCSGLGLDLRAICAGAHAL

**VDSAHA**

peptide --------------------VDSA-----HA----------------------

papillosin -----------GFWKK----VGSAAWGGVKAAAKGAAVGGLNA----LAKHIQ

centrocin 1a -----------GWFKKTFHKVSHAVKSGIHAGQRGCSALGFDLRGACAAAHAL

centrocin 2 -----------SWFSRTVHNVGNAVRKGIHAGQGVCSGLGLDLRAICAGAHAL

halocyntin ------------FWGHIWNAVKRVGANALHGAVTGALS---------------
